# Cardiac Angiosarcoma

**DOI:** 10.1155/2011/340681

**Published:** 2011-09-29

**Authors:** Monique Esteves Cardoso, Leonardo Secchin Canale, Rosana Grandelle Ramos, Edson da Silva Salvador Junior, Stephan Lachtermacher

**Affiliations:** ^1^Unidad de Vigilancia Intensiva de Cardiología Clínica, Instituto Nacional de Cardiología de Laranjeiras, 22.240-006 Rio de Janeiro, RJ, Brazil; ^2^Departamento de Cirugía Cardiovascular, Instituto Nacional de Cardiología de Laranjeiras, 22.240-006 Rio de Janeiro, RJ, Brazil; ^3^Departamento de Patología, Instituto Nacional de Cardiología de Laranjeiras, 22.240-006 Rio de Janeiro, RJ, Brazil; ^4^Departamento de Oncología Torácica, Instituto Nacional do Câncer, 20.230-130 Rio de Janeiro, RJ, Brazil

## Abstract

Despite cardiac metastases are found in about 20% of cancer deaths, the presence of primary cardiac tumors is rare. Most primary tumors are benign, and malignant tumors comprise about 15%. We report a 21-year-old man with fever, dyspnea, and hemoptysis that was diagnosed with angiosarcoma of the right atrium and pulmonary metastasis. Patient was submitted to surgical tumor resection without adjuvant therapy and died four months after diagnosis.

## 1. Introduction

Sarcomas are the main malign primary heart tumor [1], with higher prevalence in men between the 3rd and 4th decades of life, located predominantly in the right atrium. As benign lesions, the clinical presentation of malignant cardiac tumors depends on location and not the histological type. Angiosarcoma is the most common sarcoma, with high incidence of metastasis, poor prognosis, and therapy without consensus [2].

## 2. Case Report

After approval by the Ethics and Research Committee, we describe the case of a 21-year-old man with hemoptysis, fever (38°C), dyspnea, and weight loss not quantified, with any changes in the physical examination. The symptoms were treated as community acquired pneumonia, with partial improvement after antibiotic therapy. After recurrence of symptoms, chest X-ray showed new hypotransparency juxtaposed to the heart area. Magnetic resonance imaging (MRI) confirmed a large heterogeneous solid mass measuring 12 × 10 × 9 cm, located above the right atrium (RA) and superior vena cava (SVC) with extrinsic compression of the joint without cleavage plane. Evidenced still small lesion measuring 3 cm, capturing contrast, in the posterior segment of upper lobe of the right lung, a calcified nodule measuring 1 cm, not capturing contrast, in the middle lobe and residual pleural effusion. The electrocardiogram (ECG) showed sinus tachycardia and nonspecific repolarization changes. In the National Cancer Institute, patient underwent transthoracic biopsy, with diagnosis of solitary fibrous tumor. 

**Figure 1:**
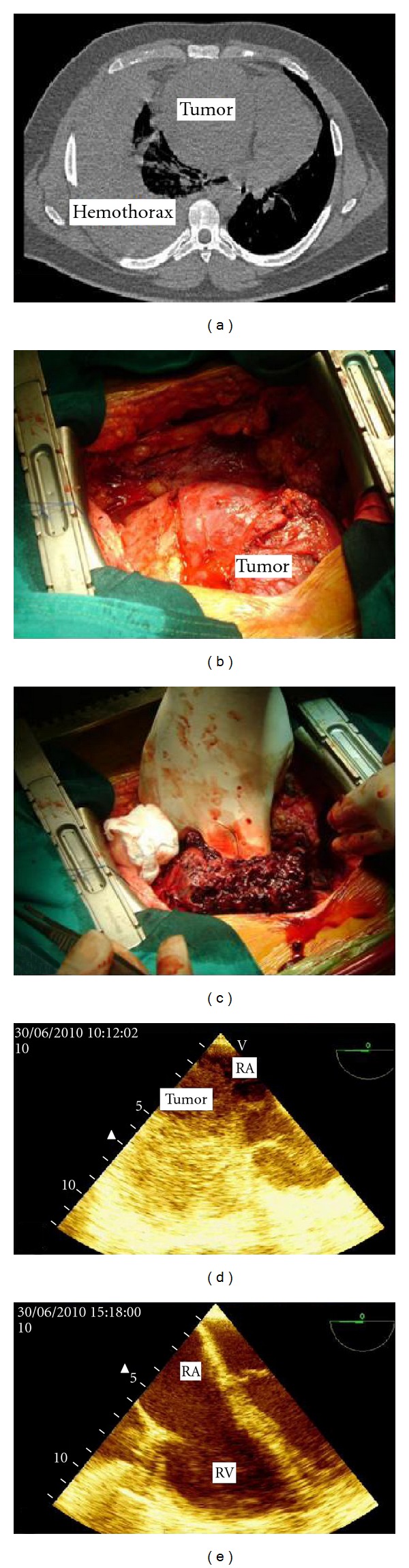
(a) Chest CT; (b) Tumor in RA displayed sternotomy (c) extensive necrosis and hemorrhage of the tumor to manipulation; (d) peroperative transesophageal echocardiogram, showing compression of RA, resolved after resection of the tumor (e) RA: right atrium; RV: right ventricle.

During hospitalization, the patient developed massive right hemothorax, diagnosed by Computed Tomography (CT) scan without contrast, as shown in Figure 1(a), where we can also see the heart tumor. One liter of blood was eliminated after thoracic drainage. He underwent surgical resection 7 months after onset of symptoms. The mass was located in the pericardium covering the access to vena cava and great vessels (Figure 1(b)). Thus, the use of cardiopulmonary bypass (CPB) was via the arteriovenous cannulation of the femoral vessels. There was no invasion of the diaphragm, pleura or pulmonary hilum. The highly vascularized mass with necrotic material was adhered to the anterior wall of RA, extending SVC, aorta, and right ventricle (RV) (Figure 1(c)). The anterior wall of the RA was resected up to the limits of SVC and the tricuspid valve. Reconstruction was performed with bovine pericardial patch. The tumor was removed without residue, without ventricular resection. The pulmonary nodule showed signs of recent hemorrhage, and wedge resection was performed. The total CBP time was 150 minutes, without the use of cardioplegia, with only 5 minutes of aortic clamping. 

**Figure 2:**
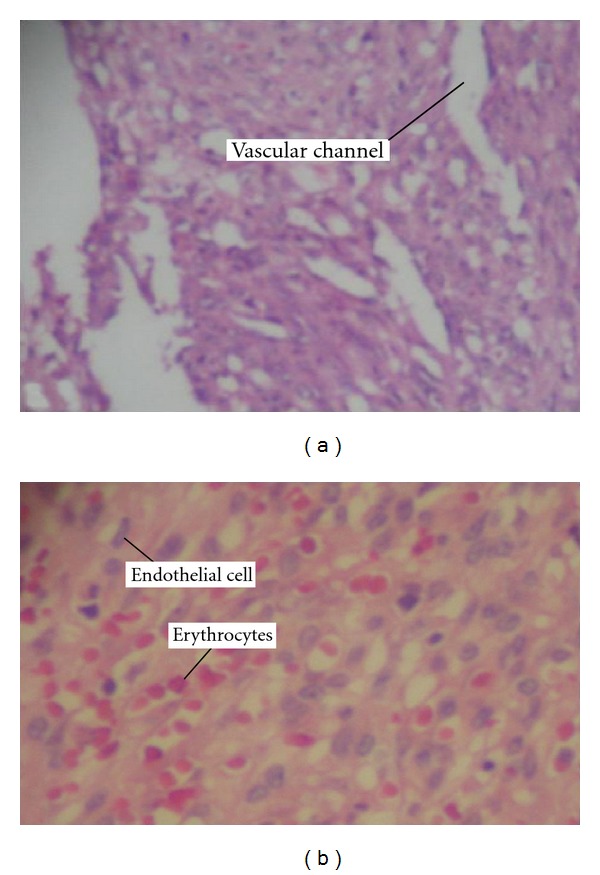
(a) Proliferation of spindle cells forming vascular channels with prominent endothelial cells, (b) vascular neoplasm with solid areas, suffused with red blood cells and endothelial cells with hyperchromatic nuclei.

The peroperative transesophageal echocardiogram showed hemodynamic compromise caused by compression of RA with resolution after resection (Figures 1(d) and 1(e)). Cytopathology of pleural fluid was negative for malignancy. Histopathology showed spongy tissue with spindle cell proliferation forming irregular vascular channels as well as varying degrees of endothelial pleomorphism and presence of mitoses and extensive areas of hemorrhage (Figures 2(a) and 2(b)). Immunohistochemistry showed immunopositivity for antivimentin, anti-CD 34, anti-CD 31, and factor VIII, with negative immune reaction for AE1 and AE3. The diagnosis was then cardiac angiosarcoma with pulmonary metastases. The limit of resection of RA is impaired.

The patient developed pulmonary sepsis and vasodilatatory shock complicated with ischemic hepatitis and renal failure requiring hemodialysis and was discharged after clinical improvement and awaiting adjuvant therapy, which was not performed. By telephone contact 4 months after surgery, we were informed by relatives that the patient was admitted at emergency department because of respiratory failure two weeks before the telephone contact. He died.

## 3. Discussion

Angiosarcoma is a disease of difficult diagnosis and reserved prognosis [3], corresponding to more aggressive histological type of sarcoma. The clinical presentation is usually delayed and depends on the local infiltration, obstruction of cardiac structures and metastases. The most common site of involvement is the right atrium, followed by left atrium, right ventricle, and left ventricle [4]. Most cases present with metastases to the lung in the moment of diagnosis [5]. Other metastatic sites are the thoracic lymph nodes, mediastinum, and vertebral column [6].

The most common symptoms include dyspnea, chest pain, heart failure (mainly right), palpitations, fever, and myalgia. Other manifestations are less frequent: pericardial effusion with or without tamponade, obstruction of the vena cava, conduction disorders [2], hemoptysis, anemia, cardiomegaly, or metastatic manifestations [4]. There may be pulmonary embolism [5] and myocardial rupture by infiltration of the tumor and necrosis [4]. On physical examination splitting of S1, heart murmurs, increased jugular venous pressure, peripheral edema, superior vena cava syndrome, hepatomegaly, and ascites can be found [6]. Signs and symptoms are nonspecific, leading to delayed diagnosis and worse prognosis [7].

Complementary exams are useful in the diagnosis of primary tumor, in assessing the extent of the disease and the presence of metastasis [6]. The ECG may reveal arrhythmias, conduction disorders, or complex of low voltage. The chest radiograph shows cardiomegaly, pulmonary congestion, or pericardial effusion. The echocardiogram may show intracavitary masses, pericardial effusion, or ventricular dysfunction. Transesophageal echocardiography has higher resolution than MRI, while it is better to identify the composition of the tissue. A chest CT is useful to highlight metastases [4].

The approach of pericardial fluid allows the analysis of cytopathology, positive in 75–87% of cases. Pericardioscopy guided biopsy has a yield of around 93–97%. A less invasive procedure is the transvenous endocardial biopsy, but it is often false negative [4]. This procedure is not recommended due to the friability of the tumor and a predisposition to bleeding, with high morbidity [6]. In our patient, the pleural fluid was negative, a transthoracic biopsy provided a false diagnosis of fibrous tumor, and definitive diagnosis was made by excision biopsy. Specific endothelial markers are found in immunohistochemical analysis.

Due to the rarity, studies to establish the treatment of cardiac angiosarcomas are scarce [2]. Surgery is the treatment of choice, dependent on the location of the tumor and metastases. Although total resection is the treatment of choice, tumor recurrence is very common [8], with a life expectancy of six months [2], depending on the presence of metastasis in clinical presentation. Chemotherapy and radiotherapy are associated with increased survival in some studies (references) and must be considered. This treatment may be indicated as adjuvant or preferential therapies, but their use is usually limited due to the poor physical condition of the patient [3]. The drugs most used in literature are a combination of cyclophosphamide, doxorubicin, vincristine, dacarbazine, mitomycin C, cisplatin, and vincristine [4]. The use of radiotherapy is limited due to cardiotoxicity [4] with high incidence of pericarditis [2].

Orthotopic transplantation could allow complete resection of the tumor, being performed in selected cases due to risk of tumor or metastatic recurrence related to immunosuppressive treatment [5].

## 4. Conclusion

The case described is consistent with those found in the literature. Cardiac tumors have nonspecific signs and symptoms. Most clinical presentation reflects hemodynamic impaired of local attempt. Diagnosis is established by image and histology analysis of the tumor. Prognosis is reserved with scarce evidence. The surgery has brought relief of symptoms of the disease and local control. The patient survival of 11 months was already expected.
